# What do IPAQ questions mean to older adults? Lessons from cognitive interviews

**DOI:** 10.1186/1479-5868-7-35

**Published:** 2010-05-11

**Authors:** Kristiann C Heesch, Jannique GZ van Uffelen, Robert L Hill, Wendy J Brown

**Affiliations:** 1 Queensland University of Technology, Institute of Health & Biomedical Innovation and the School of Public Health, Brisbane, Queensland 4059, Australia; 2 The University of Queensland, School of Human Movement Studies, Brisbane, Queensland 4072, Australia

## Abstract

**Background:**

Most questionnaires used for physical activity (PA) surveillance have been developed for adults aged ≤65 years. Given the health benefits of PA for older adults and the aging of the population, it is important to include adults aged 65+ years in PA surveillance. However, few studies have examined how well older adults understand PA surveillance questionnaires. This study aimed to document older adults' understanding of questions from the International PA Questionnaire (IPAQ), which is used worldwide for PA surveillance.

**Methods:**

Participants were 41 community-dwelling adults aged 65-89 years. They each completed IPAQ in a face-to-face semi-structured interview, using the "think-aloud" method, in which they expressed their thoughts out loud as they answered IPAQ questions. Interviews were transcribed and coded according to a three-stage model: understanding the intent of the question; performing the primary task (conducting the mental operations required to formulate a response); and response formatting (mapping the response into pre-specified response options).

**Results:**

Most difficulties occurred during the understanding and performing the primary task stages. Errors included recalling PA in an "average" week, not in the previous 7 days; including PA lasting <10 minutes/session; reporting the same PA twice or thrice; and including the total time of an activity for which only a part of that time was at the intensity specified in the question. Participants were unclear what activities fitted within a question's scope and used a variety of strategies for determining the frequency and duration of their activities. Participants experienced more difficulties with the moderate-intensity PA and walking questions than with the vigorous-intensity PA questions. The sitting time question, particularly difficult for many participants, required the use of an answer strategy different from that used to answer questions about PA.

**Conclusions:**

These findings indicate a need for caution in administering IPAQ to adults aged ≥65 years. Most errors resulted in over-reporting, although errors resulting in under-reporting were also noted. Given the nature of the errors made by participants, it is possible that similar errors occur when IPAQ is used in younger populations and that the errors identified could be minimized with small modifications to IPAQ.

## Background

Older adults who are physically active have a reduced risk of developing cardiovascular diseases, type 2 diabetes, depression and anxiety, some cancers, musculoskeletal conditions, and mobility problems [1,2]. However, older adults are typically less physically active than younger adults [1]. The burden of disease attributable to physical inactivity increases with age, with the greatest burden being found in the oldest populations [3].

Knowledge about physical activity (PA) patterns of older adults is largely based on self-report data gathered for surveillance, mostly through the use of questionnaires. Little research, however, has been conducted to examine how older adults respond to PA questionnaires developed for surveillance. Because the ability to use the same measure for surveillance in all adults, regardless of age, is critical for determining population-wide PA patterns and trends, it is important to determine whether these questionnaires are appropriate to use with older adults.

Some evidence indicates that declines in PA among older adults may be attributable to measurement errors in completing PA questionnaires [4]. Older adults may find such questionnaires challenging because they participate more in lower-intensity PA than younger adults, and lower-intensity PA is more difficult to recall than higher-intensity PA [5,6]. Older adults also tend to include unstructured PA in their daily lives, such as housework and gardening, which is difficult to recall [5,6]. Some studies suggest that older adults find it difficult to report the duration of their activities using open-ended response formats [5,7]. Errors may also arise from misinterpretation of the questions asked and/or misunderstanding of the terminology being used in these questionnaires [8].

To uncover sources of error, cognitive interviewing methods have gained in popularity over the last two decades as questionnaires have begun to be evaluated more systematically [9]. These methods can be used to ascertain whether older adults understand questions as intended by questionnaire developers and whether older adults interpret the questions in similar ways [9]. By revealing potential sources of error, cognitive interviewing methods can be used to improve the credibility of data gathered from questionnaires; therefore, they complement quantitative methods that assess the reliability and validity of questionnaires [8].

We used cognitive interviews to gain insight into how a sample of older Australian adults understood and interpreted questions from four PA surveillance questionnaires. The aim of the present study was to document participants' understanding and interpretations of questions from one of these questionnaires, the International PA Questionnaire (IPAQ). IPAQ has been used in many countries, including Australia, to assess prevalence of PA in adults aged 18-65 years [10,11] and in adults aged 18-69 years [12]. The self-report short form of IPAQ that asks participants to recall activities of the previous 7 days was used in the current study. This form of IPAQ has been recommended for international prevalence studies of adults aged ≤65 years [11]. Although it is recommended that testing of IPAQ be carried out with older adults [11], little testing of the credibility of IPAQ data has been done with this population [13,14]. Some qualitative feedback was gathered from data collectors during initial validity testing with adults aged ≤65 years [11], and qualitative data were collected from adults of all ages, 20% of whom were aged ≥65 years (n = 20), as part of an examination of over-reporting of PA on the short telephone IPAQ form [14]. However, we are unaware of any systematic collection of qualitative data to uncover cognitive problems that older adults may have with responding to IPAQ questions. This study aims to fill that gap.

## Methods

### Study participants and recruitment

Participants were community-dwelling adults, aged ≥65 years, who lived in the greater Brisbane area of Australia. To be eligible, they had to report an ability to walk >100 metres without aid, so that the sample included only people who were able to report at least some PA participation. Participants were purposively selected to ensure representation of men and women of different age groups, levels of physical activity, and education levels, since these factors have been shown to influence comprehension of PA questionnaires [15]. They were recruited via flyers displayed at voluntary organisations with large numbers of older adult members, including bridge clubs, senior centres, and exercise centres for older adults. Additionally, a recruitment notice with a request to pass study information to eligible friends and relatives was circulated through emails and e-newsletters to university staff. The study protocol was approved by the University of Queensland Medical Research Ethics Committee.

### Cognitive interviews

Cognitive interviewing typically uses as its theoretical framework the question-and-answer model of questionnaire response [16-18]. One framework is Conrad's three-stage model [19]. Conrad proposes that participants move through three stages to respond to a question: 1) understanding the intent of the question (including comprehension of what information is requested and what process should be used to retrieve that information); 2) performing the primary task (conducting the mental operations required to formulate a response, including information retrieval, mental arithmetic and evaluation of a response); and 3) response formatting (mapping a response to pre-specified response options). This classification of problems allows for a more reliable and objective interpretation of the data than when qualitative data arising from cognitive interviewing are coded without criteria [19]. The interview protocol of this study was developed to document participants' process through these three stages. Cognitive interviewing techniques that were used included 1) "concurrent think-aloud," in which respondents were asked to "think aloud" when answering questions and 2) probing, using structured and unstructured questions [16,20].

### Data collection protocol

Participants were mailed a questionnaire addressing socio-demographic and health-related characteristics and an informed consent form, which they completed and submitted at the start of the study interview. The interviews, face-to-face and semi-structured, were conducted in participants' homes or at other convenient locations, in accordance with participants' wishes. Two members of the research team with doctoral degrees in physical activity and health-related fields and with experience in qualitative interviewing (KH, JvU) alternated in serving as the primary interviewer. KH, JvU, and RLH, a graduate student in physical activity and health, alternated in serving as a second interviewer. The second interviewer was responsible for operating a voice recorder, observing and noting non-verbal signals from participants, taking notes useful for tape transcriptions, and conducting additional probing as warranted. During the interview, participants responded to questions from four PA questionnaires: the Behavioral Risk Factor Surveillance System (BRFSS) [21], Active Australia [22], the Physical Activity Scale for the Elderly (PASE) [7], as well as IPAQ [11]. A computer-based random order generator was used to assign the order in which the four questionnaires were presented. This allowed us to guarantee that similar numbers of participants would receive each of the 24 possible combinations of questionnaire order. In order to decrease any bias resulting from the order of questionnaires, participants were instructed, before the start of each new questionnaire after the first one, to respond as if they had not already responded to similar questions.

The interview began with a general introduction to the interview process and the building of rapport with participants. The primary interviewer then posed as an interviewee to demonstrate the "think aloud" process by reading out loud an example question about diet and then formulating an answer, speaking aloud her thoughts as she did so. Participants then were asked to use this technique to answer questions from the PA questionnaires. When participants did not adequately explain how they developed a response about a PA domain, the primary interviewer asked follow-up questions to probe for more information. These included comprehension-type questions (*What activities are you including in your answer?*) and questions to ascertain how the primary task was being performed (*How did you decide on your answer?*). Unscripted probing to clarify participants' responses was also used. Participants completed their response to each question by writing their answers on questionnaire answer sheets. Any problems they had with using the response format were noted.

During a refreshment break, the second interviewer measured participants' height and weight, using standard procedures. These measures were used to compute body mass index as kg/m^2^. The interview ended with the distribution of a $20 gift voucher. As soon as possible after the interview, the two interviewers, together, made field notes to begin the analysis process.

### International Physical Activity Questionnaire (IPAQ)

The self-report short form of IPAQ is a 7-item measure of four domains of activity: vigorous-intensity PA (defined as *activities that make you breathe much harder than normal*), moderate-intensity PA (defined as *activities that make you breathe somewhat harder than normal*), walking and sitting. For each activity domain, examples are provided to indicate that participants are to report activities of work, leisure-time, house and garden work, and transportation. Participants report frequency (days *during the last 7 days*) and duration (minutes/hours *usually spent on one of those days*) of their vigorous-intensity PA (VPA), moderate-intensity PA (MPA), and walking. Only sessions of activity lasting at least 10 minutes are to be reported. Participants also report the total time that they spend *sitting on a week day*, *during the last 7 days*.

IPAQ was initially developed and validated in adults aged 18-65 years from 12 countries [11], although two subsequent studies, one in Belgium [14] and one in South Africa [13], included adults over aged 65 years in their validity and reliability analyses. Validity testing has included common quantitative methods, most notably concurrent comparisons with objective measures and with other questionnaires [11,13,23-29]. In the initial 12-country validation study, the self-report short forms of IPAQ were found to have acceptable one-week test-retest reliability for PA (pooled Spearman's ρ = .75) [11]. Criterion validity for PA items on the short forms, as measured against an accelerometer, was also acceptable (pooled Spearman's ρ = .30), as it was similar to that reported for other self-report measures [11]. Estimates for sitting time on a weekday were examined with a subsample from the 12 countries [27]. Time spent sitting on a weekday had acceptable test-retest reliability (Spearman's ρ range: .62-.96), and criterion validity, as measured against an accelerometer, was also acceptable (pooled Spearman's ρ = .34). Single-country studies, however, indicate that IPAQ may result in over-reporting of PA [14,23-25,28]. The one study exclusively of older adults, a South African study of adults aged 62-70 years [13], found the specific PA domains measured in IPAQ to have adequate criterion validity when tested against an accelerometer, although total energy expenditure did not. Moreover, test-retest reliability was found to be low (Spearman's ρ = .54 in men and .60 in women) compared with estimates found in the initial 12-country study.

### Data management and analysis

Interviews were audio-recorded and transcribed. Standard qualitative methods were used for coding the data. The data in each transcription were first coded into activity domains (VPA, MPA, walking, sitting) and imported into NVivo 8 qualitative analysis software (QSR International, Melbourne, Australia). Data within each domain were next coded into the understanding, performing the primary task, and response formatting stages. In light of suggestions by Conrad [19], data for each activity domain were coded to reveal problems within each of these stages.

For the current study, only IPAQ data were analyzed. The data from the 15 participants who completed the IPAQ as the first questionnaire during the interview were coded first. Three researchers (KH, JvU, RLH) jointly developed initial themes identified within each stage, using these data. Next, data from the 26 participants who completed IPAQ as the second (n = 14) or third (n = 12) questionnaire were coded, using the initial themes and developing additional themes as they unfolded. To ensure replicability of findings across these 26 transcripts, initial coding of each transcript's data was performed by two members of the research team (KH, JvU, RLH). Discrepancies between coders were discussed in team meetings, and consensus was used to determine the final coding. The team concluded that saturation within themes had been reached after transcripts from these 26 participants had been coded, and thus the coding process ended after the coding of 41 transcripts. Next, KH reviewed all themes, merged those which overlapped, and then summarized the findings. JvU and RLH reviewed the summary and confirmed that it represented the data. For the final step, a researcher who was not included in the data collection or analysis procedures (WJB) reviewed the coding, themes, and summary report to confirm the trustworthiness of the conclusions drawn about the data collected.

## Results and Discussion

Table 1 presents sample characteristics. Both men and women were well-represented in the sample. They ranged in age from 65-89 years (mean = 72.9 years, SD = 5.9), and both low and high levels of education were represented. Also of note is that 80% of participants reported good/very good health, and the remaining 20% reported excellent health. Participants also had good physical functioning (median score on SF-36 physical function scale score = 90.0, range = 40-100) although 7% reported an inability to do exercise other than walking; 12% reported a limited ability to walk 500 meters; and 20% reported a limited ability to walk 1 km.

**Table 1:** Demographic characteristics of participants

	**Men (n = 19)**		**Women (n = 22)**		**Total (N = 41)**	
	
	**%**	**n**	**%**	**n**	**%**	**n**
Age, years						
65-69	21.1	4	36.4	8	29.3	12
70-74	42.1	8	36.4	8	39.0	16
≥75	36.8	7	27.3	6	31.7	13
Country of birth						
Australia	68.4	13	50.0	11	58.5	24
Other English-speaking country	26.3	5	36.4	8	31.7	13
Non-English-speaking country	5.3	1	13.6	3	9.8	4
Education						
No tertiary education	10.5	2	13.6	3	12.2	5
Certificate or trade	36.8	7	31.8	7	34.1	14
University degree or high	52.6	10	54.5	12	53.7	22
Employment						
Employed	5.3	1	13.6	3	9.8	4
Retired/not employed	94.7	18	86.4	19	90.2	37
Income management						
Easy	52.6	10	27.3	6	39.0	16
Not too bad	42.1	8	45.5	10	43.9	18
Difficult/impossible	5.3	1	27.3	6	17.1	7
Marital status						
Married/common-law marriage	73.7	14	50.0	11	61.0	25
Not married	26.3	5	50.0	11	39.0	16
Body mass index (kg/m^2^)						
18.5 to <25 (healthy weight)	42.1	8	40.9	9	41.5	17
25 to <30 (overweight)	47.4	9	40.9	9	43.9	18
≥30 (obese)	10.5	2	18.2	4	14.6	6

Participants reported a variety of physical activities, with some reporting only light activities, like "casual" walking, and others reporting vigorous sports as well (Table 2). For VPA, MPA and walking, participants reported transportation activities, house and garden work, and leisure-time activities, namely exercise or sports activities. For MPA and walking, some also reported caring responsibilities, including caring for children or a dog. For sitting activities, all reported leisure-time activities, mostly reading, watching television, and eating meals. Few participants reported any work activities because most were retired.

**Table 2:** Activities asked for and activities reported by participants (n = 41)

	**Vigorous activity**	**Moderate activity**	**Walking**	**Sitting**
	
Description of activities that IPAQ* asks for	*like heavy lifting, digging, aerobics, or fast bicycling*	*like carrying light loads, bicycling at a regular pace, or doubles tennis. No walking*.	*at work and at home, walking to travel from place to place, and any other walking solely for recreation, sport, exercise, or leisure*	*on a week day, including time spent at work, at home, while doing course work and during leisure time (e.g., time spent sitting at a desk, visiting friends, reading, or sitting or lying down to watch television)*
**Activities reported by participants**				

Transport-related activities	- Walk to or around shops	- Shop	- Walk to or around shops from home or from parked car	- In car - On plane
	- Walk to gym		- Walk to gym	
			- Walk to public transport	
			- Walk to other places (visit friends, to appointments, to voluntary job)	
		- Cycle to town		

Yard work	- Mow lawn			
		- Feed horse	- Feed horse	- Feed horse
		- Dig		
		- Trim/cut	- Cut	
	- Prune	- Prune		
	- Garden	- Garden	- Walk in garden	
		- Sweep outside		
		- Wash car		
		- Carrying buckets of water		

Housework	- Vacuum	- Vacuum		
		- Wash floors	- Wash floors	
		- Cleaning	- Cleaning	
			- Carry groceries to/from car	
			- Carry & hang loads of laundry	
			- Cook	
			- Sweep/mop	
			- Take rubbish out	
			- Make beds	
			- Move furniture	
		- General housework	- General housework	
			- Walk in house	

Exercise/sport	- Walk briskly		- Walk	
	- Jog			
	- Cycle	- Cycle		
	- Aqua aerobics	- Aqua aerobics	- Aqua aerobics	
	- Exercise classes (pump, pilates, yoga, 'gentle aerobics', senior fitness)	- Exercise classes (senior fitness)		
	- Dance			
	- Weight training	- Weight training		
	- Gym work	- Gym work		
	- Golf	- Golf	- Golf	
	- Table tennis			
		- Cricket		
		- Carry the bowls for lawn bowls	- Lawn bowls	
			- Croquet	

Other activities		- Carry grandchild	- Carry grandchild	
		- Mind young children		
		- Take dog out	- Walk dog	
				- Eat meals
				- Watch television
				- Read
				- Use computer
				- Visit friends
				- Attend meeting
				- Play bridge
				- Study

*IPAQ questions are available from the IPAQ website [32].

Most of the problems with responding to IPAQ questions were issues with understanding and performing the primary task. Only a few participants did not accurately record their responses as part of the third stage, response formatting. The cognitive problems encountered during each of these stages are described in Table 3 and discussed in detail below.

**Table 3:** Cognitive problems with responding to IPAQ identified during the cognitive interviews (n = 41)

Themes	Activities most affected
**Problems with understanding**	
Reporting activities of a "normal" or "average" week	VPA, MPA, walking
Confusion with *usual *and *on one of those days *being used together	VPA, MPA, walking
Including activities that lasted <10 minutes per session	VPA, MPA, walking
**Problems with performing the primary task**	
Not understanding what activities fit within the scope of a question*	VPA, MPA, walking, sitting
Reporting the same activities for more than one activity domain*	VPA, MPA, walking
Including the total time of an activity for which only a part of that time was at the intensity specified in the question	VPA, MPA, walking
Difficulty with determining frequency**	VPA, MPA, walking
Difficulty with determining duration**	VPA, MPA, walking, sitting
**Problems with response formatting**	
Confusion with how to use open-ended response format	VPA, MPA, walking, sitting

*See Table 2 for the activities reported for each activity type.

**See Table 4 for the strategies used to make the estimate.

### Problems with understanding

#### Reporting activities of a "normal" or "average" week

Before questions about each activity domain, IPAQ instructs participants to recall activities from the previous 7 days. However, some participants failed to understand that they were to report activities only of the previous week. Rather, they reported activities that were normally a part of their routine. One man, for example, said that his strategy for remembering the frequency of his MPA was to "just think back over an average week." This error could not be explained by questionnaire order. It was present in participants who completed the IPAQ first, in those who completed it after answering another questionnaire that asked about the previous 7 days, and in those who completed it after a questionnaire that had asked for activities of *a usual week*. Participants may have found recalling physical activity habits to be easier than recalling specific events of the previous week. Durante and Ainsworth [15] have suggested that general memories of physical activity habits can cloud memories of specific physical activity sessions.

In the original IPAQ validation study of adults aged 18-65 years [11], some participants were asked about activities during the last 7 days while others were asked about activities during a *usual *week. Data collectors reported that participants in some countries who were asked about activities in a *usual *week opted to report on activities in the last 7 days, because they had difficulties understanding what was meant by a *usual *week. Based on our results, it would appear that some older people have the opposite problem; they reported on a *usual *week, even though they were asked about the last 7 days.

For questions about the duration of activities, the recalling of activities of an "average" week in this study may further be explained by the questionnaire's instructions. Participants were asked to recall activities done during the previous 7 days in questions about activity frequency, but not explicitly in the follow-up questions about duration. In addition, the term *usually spend *occurs in the duration questions. Therefore, some participants may not have understood that the *previous 7 days *criterion applied to the duration questions. In discussing the duration of her MPA, one woman said, "Probably half an hour per day, I'd say. It depends on different weeks."

#### Confusion with *usually *and *on one of those days *being used together

Another source of confusion for participants was the coupling of the terms *usually *and *on one of those days *in the duration questions. This coupling created cognitive dissonance in the minds of some participants. One of the women, for example, summed up her confusion by saying:

'Usually' is something that I understand to be 'everyday,' but how can you be usually 'on one those days'? So you have to pick one day. So that is a bit contradictory to me. No, I can't answer that.

#### Including activities that lasted less than 10 minutes per session

In the introductory instructions, IPAQ asked participants to report activities lasting ≥10 minutes per session. Because this instruction immediately precedes the frequency questions and there is no similar instruction just before subsequent duration questions, participants may have perceived that the 10-minute criterion pertained only to the frequency questions. When asked for duration of activities, participants often included activities lasting less than 10 minutes per session. For example, one man, in considering the minutes he spent walking on a normal day, commented:

The question is not how much time I spend in this 10 minutes or longer lumps, but how much time I spent walking, which could include the 2-minute walk...and a lot of shorter than 10-minute walks. I don't know if you're after the 10 minutes, or longer walks. Who on earth would make such a questionnaire?

Participants such as this one, who did not apply the 10-minute criterion to the duration questions, had many more activities to include in their calculations of duration, so the task became more difficult, particularly for calculating the duration of MPA and walking. Similarly, in a Belgian sample of adults of all ages (20% aged 65+ years), Rzewnicki et al. [14] found that participants who completed the self-report *previous 7 days *telephone form of IPAQ often did not consider the 10-minute criterion as an absolute cut-off, particularly when asked about MPA or walking.

### Problems with performing the primary task

#### Not understanding what activities fitted within the scope of a question

A problem for most participants was deciding which of their activities fell within the scope of an activity domain. Some participants were unsure what to include in questions about VPA and MPA because they could not judge the intensity of their activities (see Table 2 for activities included as VPA and as MPA). One woman said, "What I'm not sure about is whether pilates is considered moderate or, uh, vigorous." Other women questioned whether certain household activities were vigorous (e.g., "what about the vacuuming or washing the floor?") or moderate (e.g., "like sweeping?"). A few participants stated that they had not done any VPA but chose to report as VPA, nevertheless, activities that they did do. For example, one woman explained the response she gave to questions about VPA duration by saying, "I'd just say for physical activities I'd do about 2 hours a day. Okay, well, I did physical activities, but not the type you're referring to."

Another difficulty concerned how participants assessed the intensity of activities given as examples of VPA and MPA. Some participants perceived that some of the examples were not consistently performed at the intensity described in the question, or that the levels of intensity required by some examples differed from the levels of intensity required for other examples. Some participants who perceived these difficulties opted to report on all activities they did that were listed as examples; others did not. The most problematic MPA example perceived by participants was *carrying light loads*. Often, participants questioned whether carrying groceries counted. One woman wondered aloud whether she should include "carrying shopping out from the garage" because "it doesn't always make me breather harder." One man said he did not know whether he should include carrying "my [lawn] bowls container, that's got four heavy bowls in it, and I carry that for 100 yards" because "what I do with my bowls is nothing like playing doubles tennis, " which was one of the other MPA examples.

A related issue was that a few participants understood the examples to be the only activities about which they were to report. One man said that he did not do any MPA because he did not do any of the activities offered as examples: "I don't play tennis anymore. The last time I played tennis I finished up in hospital...and so tennis is out. Bicycling, I don't ride bikes. Light loads, not really." One woman, in addressing the sitting question, excluded mealtimes from her answer because "it doesn't say sitting down over a meal."

Some of these difficulties with the VPA and MPA questions were also revealed in reliability and validity testing of the IPAQ in younger adults in 12 countries [11]. As part of that study, qualitative data revealed that participants had difficulty in distinguishing between VPA and MPA and in understanding the relevance of examples provided for these activity domains [11]. In the Belgian sample of adults of all ages, problems with the evaluation of intensity were also identified [14]. Thus, these problems do not seem to be age related.

Evaluating which activities fell within the scope of the walking and sitting domains was also problematic (see Table 2 for activities included as walking and as sitting). Some participants reported "casual" start-and-stop walking around the house or garden for the walking domain, and some included all activities that required movement. One woman wondered aloud whether to include her aqua-aerobics class as walking. She decided to include it. Another woman questioned whether she should only report "fast walking." Concluding that all walking should be included, she reported walking in shops and around her office at work as well as her walking for exercise. Others, however, reported only purposeful walking for exercise or for transport. Data gathered about the sitting question revealed that some participants were unclear which activities not listed as examples of sitting were to be included. Some participants reasoned that lying down activities should not be included while others concluded that naps and lying down while not reading or watching television should be counted as sitting. One woman asked, "I mean, is sitting down not standing up?"

#### Reporting the same activity for more than one activity domain

Many participants reported the same activity, usually walking, in two or three activity domains. With no instruction to exclude walking from VPA, participants included walking briskly for exercise or transport in the VPA domain as well as in the walking domain. Likewise, activities that involved carrying a light load were often reported in both the MPA and the walking domains. Sports that require walking, such as golf and lawn bowls, were sometimes counted as VPA and/or MPA, and also walking. Activities that included both VPA and MPA components (e.g., cycling up and down hills, exercise classes, house and garden work) were sometimes counted within both the VPA and MPA domains.

#### Including the entire time of an activity for which only a part of that time was at the intensity specified in the question

The time from the beginning of an activity until its conclusion was usually reported, even when the level of intensity specified in the question was not present throughout the entire time period. For example, a 1-hour exercise class that included periods of stretching, aerobic exercise, weight training, and warm up and cool down periods was often counted as one hour of VPA and/or MPA. Participants who walked for exercise or transport often included the total time of the walks, not accounting for breaks to sit down or to stand. One man reported a 3-hour "shopping expedition" for the MPA domain because it required carrying light loads. When probed about the expedition, he explained that the start time of the 3-hour expedition was "about when we leave here [home]...by car."

#### Difficulty with determining frequency

Experiencing difficulty in recalling how often they had done some activities, participants enlisted various strategies for calculating or estimating the number of days that an activity was done (Table 4). Our qualitative data indicated that VPA was the easiest for participants to recall, particularly when the activities were exercise or sporting activities performed regularly. Recall of the frequency of MPA and walking activities was often more challenging. Some participants had to use "guess work" or use "a little bit of an estimate" to respond to the questions about these domains. One strategy used for recalling MPA and walking was assuming that these activities were a requirement of daily living. One man said about walking, "It would have to be 7 days; otherwise, you'd be dead, wouldn't you?" A woman reported she did MPA every day because "I'm always moving and active and walking: I can't sit still very long." Only two strategies acknowledged that the question asked for activities performed in the previous 7 days: 1) counting the number of days that an activity was done in the last 7 days (the method likely intended by IPAQ developers) and 2) counting the number of days that the activity was not done and subtracting that number from seven.

**Table 4:** Strategies participants used for determining frequency and duration of their activities

Strategies	Activities most affected
**For determining frequency**	
Count the number of days the activity was done in the previous week	VPA, MPA, walking
Subtract the number of days that the activity was not done in the previous week from 7 days*	VPA, MPA, walking
Report the specific or minimum number of days the activity was performed in a "normal" or "average" week	VPA, MPA, walking
Estimate or guess based on a "normal" or "average" week	MPA, walking
Report the activity was done daily because it was required as part of living	MPA, walking
**For determining duration**	
Sum duration of activities of the previous week, and then average across the days the physical activity was done	VPA, MPA, walking, sitting
Sum duration of the activity that was done the most often during the previous week*	VPA, MPA, walking
Estimate average time spent sitting during a typical day in a "normal" or "average" week	Sitting
Estimate the proportion of a typical day spent sitting in a "normal" or "average" week	Sitting
Subtract from 24 hours the time spent sleeping and doing other types of activities in a "normal" or "average" week, to arrive at time spent sitting	Sitting
Sum minimum or maximum amount of time spent doing the activity on a typical day in a "normal" or "average" week*	VPA, MPA, walking
Select a particular day from the previous week (e.g., the easiest day to remember, the most enjoyable day, the day that the most activity was performed) and use one of the following strategies to compute time on that day	VPA, MPA, walking, sitting
• Recall typical duration of the activity	VPA, MPA, walking
• Guess the duration	MPA, walking, sitting
• Recall typical duration of some activities and guess the duration of others	MPA, walking, sitting
Combine two or more strategies because time in some activities is known and time in other activities is not known	VPA, MPA, walking, sitting
Do not provide an answer because unable to develop a strategy to answer question	Walking, sitting

*A strategy reported by only a few participants

#### Difficulty with determining duration

Calculating the duration of activities within each domain was more challenging than recalling their frequency. Likewise, Rzewnicki et al. [14] identified difficulties in summing minutes or hours over a day, particularly for walking and MPA, in adults of all ages. Table 4 lists the strategies that participants used for formulating a response to duration questions. Taking an average was a method used across all domains. Doing so required a number of calculations, and errors in calculations were sometimes made. For example, one woman averaged her time spent sitting on a weekday as follows:

It'd be about 8 hours in front of the TV almost three nights...and then it's only another half hour the other four nights, that's 2 hours. So that's about 8 and 4 are 12 hours, plus I suppose sitting...eating tea, eating your meals. I suppose you've got to take another hour a day [for those activities] there, 7 days. What are we up to? That's about 12 and 7 [hours], 19 and then at the computer a couple of hours I suppose, 21 [hours]...So if we round it up to 22 that'd be about all. So I'd put down 22 hours. How much time? Per day? Am I going to divide it by seven? Three hours a day, okay?

A few participants interpreted the duration question to refer to the activity done on the greatest number of days of the previous week. Giving a carefully thought-out response, one man reported 20 minutes of weight lifting for MPA because he did weight lifting more days the previous week (3 days) than his other activity (1 day).

To calculate sitting time, participants developed strategies not used for the other domains (Table 4). It was common for participants to make an estimation of the time spent sitting on a typical day of a "normal" or "average" week. Some participants estimated the proportion of such a day spent sitting. One woman reported, "I've worked out that I'm up [out of bed] for 15 hours most days; two thirds [of that time] is 10 [hours spent sitting]." Others subtracted (accurately or not) the time doing other things during a typical day to arrive at the time spent sitting. One man said, "24 hours in a day. I am sleeping from about 11 to about 5, so that's about 6 hours gone, 18 hours left of the day. I am doing about 3 [hours] of exercise. Would you believe...I spend 12 hours a day sitting down?"

The phrase *on one of those days *in the previous 7 days led some participants to understand that they were to pick a particular day. They selected the day in which they did the most activity that fitted within the domain of the question. Once they had selected the day, they developed a strategy for calculating the duration of the activity performed on that day. The most common strategies used for calculating the duration of activities done on that day were 1) recalling the typical duration of the activities (e.g., sports or exercise with pre-set durations like exercise classes or regularly scheduled walks); 2) guessing (e.g., house or garden work; walking casually around the house); and 3) estimating based on a recall of the typical duration of some activities and then guessing the duration of other activities. In recalling his walking, one man used the third strategy. He reported, "On ONE of those days, oh crikey.... I know I walk for an hour a day at least. I just would have to have a guestimate there and say another 2 hours fiddling around."

Some participants could not come up with a strategy for reporting duration. They found the task too difficult because, they said, they did not know how much time they did activities within the domain or, because the activities varied between days, they did not know what to report for time *usually spent on one of those days*. This was mainly a problem with the walking and sitting questions. In trying to respond to the walking question, one man said, "I don't know how to answer it. I really don't. I could put 10 minutes. I could put 45 minutes. I could put 30 minutes. It just depends which day I pick." The walking and sitting questions were particularly difficult for people who reported that they did these activities throughout the day. After reading the sitting question, one woman said,

You've got to sorta think...you sit down and have your meals and you don't necessarily sit down for the same amount of time...It's not like when you're at work and you have a half hour for lunch and so you sit down and you know that's a half an hour...You can sit down to have a cup of coffee and you can spend 5 or 10 minutes having a cup of coffee...You can pick up a book and get engrossed with the book and, you know, the time goes. It's very, very hard to answer....That is in the 'too hard' basket.

### Response formatting

Only a few participants had difficulty fitting their responses into the response formats presented. One participant, reporting an hour's activity, wrote the same answer in minutes (60) and then again in hours (1). Another reported the weekly duration of an activity as one day's amount. A third wanted to describe how long he had spent on an activity with a range of times.

### Recommendations

Recognizing the value of IPAQ for international PA surveillance, in Table 5 we make recommendations to increase its usefulness for surveillance of PA in older adults aged ≥65 years. We note that previous findings indicate that some younger adults have difficulties in understanding IPAQ questions [10,11,14] or over-report their activities on IPAQ [10,14,23-25,28], findings consistent with those presented here. We acknowledge that any modifications would need to be included in versions used for younger adults also, for consistency across age groups, and that any modifications would need to be further tested before being used. We are also aware that making any changes to IPAQ may mean that the ability to track changes in PA over time may be lost, because the modifications may decrease the amount of PA reported.

**Table 5:** Authors' recommendations for improving IPAQ for older adults

1	List in the instructions at the beginning the activities that will be asked for in the questionnaire (i.e., VPA, MPA, walking, sitting).
	
2	State in the instructions at the beginning and for each activity domain that activities already reported for one domain should not be reported again for another.
	
3	State in the instructions at the beginning and for the duration questions that only activities lasting at least 10 minutes during the previous 7 days should be included.
	
4	For VPA and MPA, state in the instructions to include only the amount of time spent at the specified intensity.
	
5	For VPA and MPA, state in the instructions that the examples given are only indicators of activities which might be done at the specified intensity and that the examples are not to be used as choice-limiting checklists.
	
6	Add examples of activities relevant to older adults and clarify activities mentioned as examples in the questionnaire.
	
7	Provide instructions for the walking activities to report.
	
8	Offer strategies for determining the frequency and duration of activities.
	
9	For duration questions, clarify or revise the phrase *usually spent on one of those day*s.

We suggest that the order of the questions stay the same. Ainsworth et al. [23] have suggested that the placement of the VPA questions before the MPA questions may be responsible for the higher amounts of PA reported in the IPAQ. However, in a comparison of four PA questionnaires, Brown et al. [30] attributed the higher levels of PA found for IPAQ, in comparison to the other questionnaires, to the fact that the IPAQ examples encourage people to think about PA more broadly (i.e., PA at work, leisure, and transport) than do the other questionnaires. In the study by Brown et al., there was no indication that the order of the questions made a difference in the amount of PA reported. However, respondents did find it easier to recall more structured or routine activities, which are typically associated with sport or recreation, or walking specifically for exercise, rather than transport to and from places [31]. It might therefore be better to ask the VPA questions first, as these activities may be easiest to recall. We recommend, however, that participants be instructed at the beginning of the questionnaire that they will be asked separately about VPA, MPA, walking and sitting activities to reduce the tendency to report activities early in the questionnaires (e.g., VPA) when they should only be reported later (e.g., MPA or walking).

Other recommendations for correcting over-reporting are to clarify in the instructions at the beginning and for each activity domain that activities already reported for one domain should not be reported again for another and to state in the instructions at the beginning and for the duration questions that only activities lasting at least 10 minutes during the previous 7 days should be included. For VPA and MPA, participants could also be instructed to include only the amount of time spent at the specified intensity and to treat the examples as indicators of activity types only, not choice-limiting checklists. To augment older adults' understanding of IPAQ questions, we recommended adding examples of activities relevant to older adults (e.g., those in Table 2 for Australian older adults), and clarifying activities mentioned in the questionnaire, such as *carrying light loads *for MPA and *lying down watching TV *for sitting (e.g., is other lying down not to be included?). To decrease the reporting of short-duration, start-and-stop walking, clarification of the types of walking to report should be included, and to further decrease reporting of walking more than once, the instructions for the VPA domain should tell participants to exclude walking.

We also suggest that, in order to create more consistency across participants in how calculations are made, participants be given guidance on determining the frequency and duration of their activities. Given that most of our participants over-reported activities but that some under-reported, such guidance could reduce recall bias or, failing that, at least make this bias go in one direction so that compensating adjustments might be easier. Durante [15] suggested that for questionnaires with short referent periods, like IPAQ, questions may be more effective if they use episode enumeration techniques for gathering data. Applying this to IPAQ, participants could be told to count up their days of PA over the previous 7 days, preferably starting with the previous day and working backwards, to compute frequency, and to sum their minutes of PA over a specific day to calculate duration. Moreover, participants could be given a strategy to use for selecting the specific day. Instructing participants to recall a specific day (e.g., the last weekday prior to questionnaire completion) could help in recall of activities and result in a more consistent method of calculation. Such instruction, by drawing attention to a particular day of the past week, may also decrease some participants' tendency to recall activities of a "normal or "average" week

### Limitations

The findings of this study would likely have been different if other forms of IPAQ had been administered or other modes of administering IPAQ had been used. Also, the study created an artificial testing environment and participants may have responded differently if they had completed the questionnaires on their own. To decrease this possibility, participants were told to answer each question as they perceived it should be answered, with no guidance from the interviewers. Probing only occurred after participants had completed questions about an activity domain, and any changes participants made to their answers in response to the probing were not included in our analyses. It should also be noted that participants who completed IPAQ as the second or third questionnaire during their interview could have been influenced by questions asked in questionnaires they completed before completing IPAQ. However, this is not likely as review of the transcripts from participants who completed IPAQ as the first questionnaire revealed that all themes discussed in this paper were present in these participants' transcripts. It should also be noted that although participants represented a full range of educational levels, they were, on average, fairly well educated. It is likely that a less well educated sample would have had even more problems understanding IPAQ questionnaires than our participants.

## Conclusions

Cognitive interviews with Australian adults aged ≥65 years revealed problems with using the self-report *past 7 days *form of IPAQ. Data collected from participants' "thinking aloud" as they answered the IPAQ questions and from their responses to probing questions about their answers uncovered problems older adults may encounter when completing IPAQ. These included difficulty in understanding the intent of the questions, in recalling the information requested, and in making the calculations required to perform the task. For most participants, errors resulted in over-reporting, although for a few participants errors resulted in under-reporting. Participants experienced more difficulties with the MPA, walking and sitting questions than with the VPA questions. The question that asked for the duration of sitting time on a weekday required strategies different from those used to answer questions about the other activity domains. Our findings indicate that caution is warranted in administering IPAQ to adults aged ≥65 years. It is possible that the errors identified could be minimized by modifying IPAQ, and we have suggested possible ways to do this. Future research, however, is required to test whether these errors occur when IPAQ is used in other populations (e.g., younger age groups and less educated older adults), and to test whether these changes result in improvements to the accuracy of the data collected with IPAQ from both older adults and younger adults.

## Competing interests

The authors declare that they have no competing interests.

## Authors' contributions

KCH and JvU developed the study design, interviewed the participants, and analyzed and interpreted the data. KCH developed the data coding and analysis process and drafted the manuscript. JvU and WJB conceived the study, and WJB also participated in the analysis and interpretation of the data. RLH participated in conducting the interviews and analyzing and interpreting the data. All authors participated in revising manuscript drafts and in reading and approving the final manuscript.
